# Systematic review of the health and social determinants and outcomes of home cooking: protocol

**DOI:** 10.1186/s13643-015-0033-3

**Published:** 2015-03-28

**Authors:** Susanna Mills, Martin White, Shannon Robalino, Wendy Wrieden, Heather Brown, Jean Adams

**Affiliations:** Institute of Health and Society, Newcastle University, Baddiley-Clark Building, Richardson Road, Newcastle upon Tyne, NE2 4AX UK; Centre for Diet and Activity Research (CEDAR), MRC Epidemiology Unit, University of Cambridge School of Clinical Medicine, Box 285 Biomedical Campus, Cambridge, CB2 0QQ UK; Human Nutrition Research Centre, Institute of Health and Society, Newcastle University, M1.151 William Leech Building, Medical School, Framlington Place, Newcastle upon Tyne, NE2 4HH UK

**Keywords:** Public health, Cooking, Diet, Obesity

## Abstract

**Background:**

The United Kingdom (UK) and wider world are experiencing an obesity epidemic, with lower socio-economic groups disproportionately affected. Dietary quality is also socio-economically patterned, with an estimated quarter of observed inequalities in UK mortality due to inequalities in diet. Food preparation and eating patterns clearly have an impact on dietary intake and hence health. A growing body of evidence indicates that out of home food consumption and eating ready meals may be associated with negative outcomes. However, to date no systematic reviews have assessed the health and social determinants and outcomes of home cooking. Here, home cooking refers to the combination of actions required for preparing hot or cold foods at home, including combining, mixing and often heating ingredients.

**Methods/Design:**

A systematic review of peer-reviewed literature on home cooking will be undertaken. Studies will be considered for inclusion if they present qualitative or quantitative data on participants from high/very high human development index countries, including all relevant study designs. No language or date of publication restrictions will be applied. Determinants will be considered as factors that influence behaviour and outcomes as potential advantages and disadvantages of engaging in home cooking. Electronic databases of peer-reviewed journal articles covering health, psychology, social sciences and consumer practices will be searched. Published postgraduate theses will also be considered for inclusion. Additional strategies to identify relevant studies will be used, such as citation searches of included articles, evaluation of references from relevant reviews and included articles and the ‘related/similar to’ function found in certain databases. Two independent researchers will be involved in literature screening (10% at first screen and 100% at second screen), data extraction and quality appraisal. Studies included in the review will be analysed by thematic synthesis and narrative synthesis, as appropriate for the nature of the data retrieved.

**Discussion:**

This review will provide key empirical evidence to inform the development of recommendations for public health policy makers and practitioners to encourage healthier home food preparation, thereby impacting on dietary-related health.

**Systematic review registration:**

This protocol has been registered with the PROSPERO international prospective register of systematic reviews, reference CRD42014013984.

**Electronic supplementary material:**

The online version of this article (doi:10.1186/s13643-015-0033-3) contains supplementary material, which is available to authorized users.

## Background

The United Kingdom (UK) and wider world are experiencing an obesity epidemic, with lower socio-economic groups disproportionately affected. Dietary quality is also socio-economically patterned, with an estimated quarter of observed inequalities in UK mortality due to inequalities in diet [1]. Food preparation and eating patterns clearly have an impact on dietary intake and hence health. However, the relationships between home cooking and both the quality of dietary intake and the morbidity associated with poor diet remain unclear. Here, home cooking refers to the combination of actions required for preparing hot or cold foods at home, including combining, mixing and often heating ingredients. Home cooking practices are individualised, culturally embedded and shaped by societal changes, such as decline in family mealtimes and increasing availability of pre-prepared meals [2]. There is evidence of a decline in time spent cooking at home since the mid-twentieth century in developed countries (such as the United States of America [3], and Germany [4]). This has been accompanied by a paradoxical increase in public interest in food, illustrated by rising popularity of television cookery shows, cookbooks and celebrity chefs [5]. The apparent decline in home cooking has occurred in parallel with rising obesity levels, such that overweight and obesity have become the norm [6]. The average UK adult diet also contains excess saturated fat, insufficient fibre, too much added sugar, inadequate amounts of omega-3 fatty acids and too much salt compared to a diet recommended for the avoidance of non-communicable chronic diseases [7,8].

There is a growing body of evidence to suggest that a correlation exists between out of home food consumption (for example [9]) and eating ready meals (for example [10]) and poor health outcomes. To date, no systematic reviews have assessed the health and social determinants and outcomes of home cooking. Here, ‘health’ factors include issues such as dietary quality and body weight. ‘Social’ factors include variables such as extent of cooking skills, socio-economic status and capacity for leisure activities. A full understanding of the relationships between cooking and dietary-related health is crucial, since cooking skills form a cornerstone of UK government policy on obesity reduction [11], and in accordance with Medical Research Council guidance [12], insights will provide the foundations for developing complex interventions promoting healthier home eating.

## Methods/Design

### Protocol and registration

This protocol has been registered with the PROSPERO International Prospective Register of Systematic Reviews [13] reference CRD42014013984 and reported adhering to the Preferred Reporting Items for Systematic Review and Meta-Analysis Protocols (PRISMA-P) 2015 statement [14]. The final review will be reported according to recommendations from the Preferred Reporting Items for Systematic Reviews and Meta-Analyses (PRISMA) guidelines [15].

### Objective

This systematic review will present a comprehensive summary of current evidence regarding the health and social determinants and outcomes of home cooking. It will help inform recommendations for policy makers, researchers and practitioners for the development of healthy eating interventions and areas for further research.

### Search strategy

Standard searching methods will be employed to conduct this review, according to guidance from the York Centre for Reviews and Dissemination [16], the Cochrane Collaboration [17], and the Evidence for Policy and Practice Information and Co-ordinating Centre (EPPI-Centre) at the Institute of Education, London [18]. The following electronic databases of peer-reviewed journal articles will be searched for relevant studies: MEDLINE; Scopus; Web of Science; PsycInfo; Applied Social Science Index and Abstracts (ASSIA); Business Source Premier; CAB Abstracts; CINAHL; Evidence Based Medicine Reviews (EBMR) - Cochrane Central Register of Controlled Trials, Cochrane Database of Systematic Reviews and Database of Abstracts of Reviews of Effects; Embase; Education Resource Information Centre (ERIC); Health Management Information Consortium (HMIC); International Bibliography of the Social Sciences (IBSS); Public Affairs Information Service (PAIS) International; Social Services Abstracts and Sociological Abstracts. The National Centre for Biotechnology Information (NCBI) PubMed database will also be searched. A sample search strategy for MEDLINE is shown in Additional file 1.

Additional peer-reviewed published studies will be identified by evaluating references cited in relevant reviews and the references of literature meeting the inclusion criteria for this review. Citation searches of included articles will be undertaken using the Science and Social Science Citation Indices. Further relevant studies will also be identified using the ‘related/similar to’ function found in certain databases. Postgraduate theses satisfying the inclusion criteria will be incorporated into the review. The obligation for peer-review will be satisfied if the thesis has been submitted and met the standards required by the student’s examination board. Other grey literature (texts not published in peer-reviewed journals), such as local project reports, will be excluded. The resources required to ensure comprehensive coverage of such material is beyond the scope of this review.

The search strategy will be developed and enhanced with the assistance of an information scientist specialising in medical and social sciences literature. Search strings created from designated keywords and search terms will be iteratively expanded according to the results generated from initial searches. A search diary will be employed to record the finalised generic search strategy, databases searched, details on the studies identified and the decision regarding their suitability for inclusion. Initial scoping searches have been conducted and used to identify a realistic timeframe and suitable milestones for the execution of the full search and review report.

### Selection criteria

This review will address ‘home cooking’, defined as the practices and skills for preparing hot or cold foods at home, including combining, mixing and often heating ingredients. It will consider, as two separate arms of the review, firstly, the health and social *determinants* of home cooking (factors that influence behaviour), such as availability of leisure time, socio-economic status and extent of cooking skills. Secondly, it will consider the health and social *outcomes* of home cooking (the benefits and disadvantages), such as dietary quality, body weight, self-esteem and financial security. Determinants and outcomes addressing food safety, or specific diseases not generalisable to the wider population, will not be included. Home cooking must be mentioned as a key focus of the study and discussed in the methods and/or results section. The definition of home will include private households and other self-catered domestic arrangements, for example university accommodation. Comparators may include alternatives to home cooking, such as consumption of takeaway meals and pre-prepared ready meals, or there may be no comparator specified.

Studies investigating home cooking for specific disease groups or physical incapacities not generalisable to the wider population, or specific dietary requirements such as for food allergies/intolerances and professional sportspeople, will be excluded. Similarly, research focussed on commercial locations such as restaurants, or providing analysis of specific dishes or food preparation techniques, or addressing cooking practices prior to the 20th century, will not be incorporated into the review. The population of interest will include child, adolescent, adult and elderly participants living in high/very high human development index countries [19]. Participants living outside these countries will be excluded, since issues encountered in areas with lower levels of development, such as smoke inhalation from cooking fires, are not necessarily generalisable to more developed nations. All studies included in the review must be peer-reviewed, with a title in English, and present qualitative or quantitative data on home cooking from observational or experimental designs. Studies designed as a discussion or a review paper will be excluded, although the reference lists will be screened for relevant primary studies.

### Study selection

#### Initial screening

An EndNote database will be used to store retrieved literature and duplicate entries will be removed, since the same literature may be identified in several different databases [20]. In any cases where more than one report type is retrieved for the same study, preference will be given to peer-reviewed information and the hierarchy of research study design [21]; however, additional details will be extracted from the different sources where relevant. Authors will also be contacted to identify whether a peer-reviewed report is available for the study, if a non-peer-reviewed version has been retrieved. In order to identify all potentially relevant studies, the titles and abstracts of articles located through the search strategy will initially be screened against the inclusion criteria for the review. Papers will be designated as either ‘not relevant’ or ‘potential’. Screening will be undertaken by the lead reviewer, and a second reviewer will screen 10% of papers, in alignment with evidence supporting the improvement of reliability and reproducibility in decision-making by involving more than one reviewer in this process [22]. Titles and abstracts generating a difference of opinion between reviewers at this stage will be included.

#### Second screening

Full texts of the literature deemed potentially suitable, or generating a difference of opinion between reviewers, will subsequently be acquired. A standardised checklist of the eligibility criteria will then be used to make a decision regarding inclusion. Second screening will be undertaken by both the lead reviewer and a second reviewer. In the event of disagreement regarding inclusion, consensus will be reached through discussion amongst the full research team. References for excluded studies and the reason for their rejection will be retained in a separate folder of the project EndNote database.

#### Data extraction

Following the identification of literature appropriate for inclusion in the review, a bespoke data abstraction framework will be used as a template for recording significant study characteristics. This information will include details, as appropriate, on: study design, number of participants, intervention and comparator, participant demographics, setting, time period, outcomes and outcome measures, perspective, analysis, results and quality appraisal. Data will be extracted by the lead reviewer and checked by a second reviewer. The data will be tabulated to create a Microsoft Excel spreadsheet summary, thus facilitating accurate comparison between studies. To ensure a comprehensive record of relevant, precise information, the abstraction tool will be piloted on a small sample of literature selected for inclusion in the review and modified as necessary. Studies similar in terms of population and recorded outcome measures will be grouped together in the summary for quality assessment and data synthesis as appropriate. According to the hierarchy of research study design, findings will be given greater preference in descending order from randomised controlled trials, cohort studies, case-control studies, cross-sectional surveys and case reports. In circumstances where insufficient details have been provided to permit complete data extraction or quality appraisal, the study authors will be contacted for further information.

#### Quality assessment

Quality appraisal will be undertaken by two researchers working independently using an appropriate tool for the type of study conducted. The quality of quantitative studies included in the review will be assessed using the Effective Public Health Project tool [23]. This resource demonstrates good inter-rater reliability, is applicable across a range of quantitative study types and is recommended by the Cochrane Public Health Group. The quality of qualitative studies will be assessed using a checklist developed by Smith *et al*. to combine items from a range of previous tools [24]. The quality of data and the data reporting will be presented with the review findings to enable a hierarchical approach to the interpretation of the results.

#### Data synthesis

It is anticipated that a diverse range of research methods will be identified within this review, and hence statistical meta-analysis is unlikely to be appropriate. In the event that several qualitative studies are identified meeting the inclusion criteria, qualitative synthesis will be undertaken through thematic synthesis. This is a three-stage process, with a first step of adding codes to the data line by line, secondly identifying core descriptive themes and thirdly providing interpretive analytical themes. This approach produces findings that directly inform practitioners [25] and is more directly relevant to policymakers and designers of interventions than methods with a more constructivist orientation [26].

The Economic and Social Research Council have developed a set of guidelines, incorporating specialised tools and techniques, and a broader framework, for the construction of a clear, reliable narrative synthesis report [27]. This framework will be used to construct a narrative synthesis of the quantitative data in four main stages:

- Constructing a novel theory of what health and social *factors* may *influence* home cooking; what health and social *outcomes* home cooking may *produce*; why and for whom.
- Creating an initial synthesis of the results of included literature.
- Investigating relationships and any associations identified within and between studies.
- Analysing the extent to which the synthesis of this data can be considered robust.

This narrative synthesis will also include the themes identified through thematic synthesis of the qualitative literature to facilitate triangulation of findings. Data from both quantitative and qualitative studies will then be collated in a tabulated summary of themes, with appropriate grouping according to study characteristics. A clear accompanying descriptive account addressing the robustness of the evidence will be provided. If quality is found to vary between studies, a sensitivity analysis will be undertaken, addressing whether the exclusion of studies with poor quality rating has an impact on the precision and overall conclusions of the review.

## Discussion

### Strengths and limitations

A strength of this review is the use of a systematic and transparent approach, employing recommended and validated methods. The review is inclusive and comprehensive by incorporating a wide range of determinants and outcomes relating to home cooking and is unbiased regarding their relative importance. Including only peer-review literature will facilitate increased validity of the review findings. The involvement of two researchers at each stage of literature screening, data extraction and quality appraisal will also increase the reliability of the conclusions drawn. This review is widely relevant to the general population and pertinent for current UK policy [11]. A limitation of the review is the complex nature of home cooking as a topic, with inherent difficulties in defining terms and producing clear conclusions and recommendations. There might be relevant papers with non-English titles which we excluded at initial screening. Similarly, there could be additional useful insights drawn from including grey literature; however, the resource requirements for this approach are beyond the scope for this review.

### Dissemination

The findings from this review will be shared with policy makers and practitioners through local stakeholder groups feeding into national organisations. Written dissemination will be achieved through a summary publication for practitioner readership and submission to a peer-reviewed journal. The results will be presented at a national conference and circulated to the general public using social media.

## Additional file

Additional file 1:
**Sample search string for Ovid MEDLINE.** Search string iteratively developed by information scientist.

## Abbreviations

ASSIA: Applied Social Science Index and Abstracts
EBMR: Evidence-based Medicine Reviews
ERIC: Education Resource Information Centre
HMIC: Health Management Information Consortium
IBSS: International Bibliography of the Social Sciences
NCBI: National Centre for Biotechnology Information
PRISMA: Preferred Reporting Items for Systematic Reviews and Meta-analyses
PRISMA-P: Preferred Reporting Items for Systematic Review and Meta-analysis Protocols
PROSPERO: International Prospective Register of Systematic Reviews
PAIS: Public Affairs Information Service
UK: United Kingdom

## References

[1] Stringhini S, Dugravot A, Shipley M, Goldberg M, Zins M, Kivimäki M (2011). Health behaviours, socioeconomic status, and mortality: further analyses of the British Whitehall II and the French GAZEL prospective cohorts. PLoS Med.

[2] Lang T, Caraher M, Dixon P, Carr-Hill R (1999). Cooking skills and health.

[3] Smith L, Ng S, Popkin B (2013). Trends in US home food preparation and consumption: analysis of national nutrition surveys and time use studies from 1965–1966 to 2007–2008. Nutr J.

[4] Möser A (2010). Food preparation patterns in German family households. An econometric approach with time budget data. Appetite.

[5] Pollan M (2013). Cooked: a natural history of transformation.

[6] Department of Health. Reducing obesity and improving diet. 2013. http://www.gov.uk/government/policies/reducing-obesity-and-improving-diet Accessed 19 Feb 2015.

[7] Bates B, Lennox A, Swan G (2010). National diet and nutrition survey: headline results from year 1 of the rolling programme (2008/2009).

[8] Swan G (2004). Findings from the latest national diet and nutrition survey. Proc Nutr Soc.

[9] Beydoun MA, Powell LM, Wang Y (2009). Reduced away-from-home food expenditure and better nutrition knowledge and belief can improve quality of dietary intake among US adults. Public Health Nutr.

[10] Lobato JC, Costa AJ, Sichieri R (2009). Food intake and prevalence of obesity in Brazil: an ecological analysis. Public Health Nutr.

[11] Department of Health. Change4Life. 2013. http://www.nhs.uk/Change4Life/Pages/healthy-eating.aspx. Accessed 19 Feb 2015.

[12] Craig P, Dieppe P, Macintyre S, Michie S, Nazareth I, Petticrew M (2008). Developing and evaluating complex interventions: the new Medical research council guidance. BMJ.

[13] University of York Centre for Reviews and Dissemination. PROSPERO: International prospective register of systematic reviews. 2013. http://www.crd.york.ac.uk/NIHR_PROSPERO/. Accessed 19 Feb 2015.

[14] Moher D, Shamseer L, Clarke M, Ghersi D, Liberati A, Petticrew M (2015). Preferred reporting items for systematic review and meta-analysis protocols (PRISMA-P) 2015 statement. Syst Rev.

[15] Moher D, Liberati A, Tetzlaff J, Altman DG (2009). Preferred reporting items for systematic reviews and meta-analyses: the PRISMA statement. BMJ.

[16] Centre for Reviews and Dissemination. Systematic Reviews: CRD’s guidance for undertaking reviews in healthcare. 2009. http://www.york.ac.uk/inst/crd/SysRev/!SSL!/WebHelp/SysRev3.htm. Accessed 19 Feb 2015.

[17] Higgins JP, Green S. Cochrane handbook for systematic reviews of interventions. 2011. http://handbook.cochrane.org/. Accessed 19 Feb 2015.

[18] Gough D, Oliver S, Thomas J (2012). An introduction to systematic reviews.

[19] Program UND (2014). Human development report 2014.

[20] von Elm E, Poglia G, Walder B, Tramer E (2004). Different patterns of duplicate publication: an analysis of articles used in systematic reviews. JAMA.

[21] Preventive Services Task Force (1996). Guide to clinical preventive services: report of the U.S. preventive services task force. 2nd ed.

[22] Edwards P, Clarke M, DiGuiseppi C, Pratap S, Roberts I, Wentz R (2002). Identification of randomized controlled trials in systematic reviews: accuracy and reliability of screening records. Stat Med.

[23] Thomas BH, Ciliska D, Dobbins M, Micucci S (2004). A process for systematically reviewing the literature: providing the research evidence for public health nursing interventions. Worldviews Evid Based Nurs.

[24] Smith KE, Bambra C, Joyce KE, Perkins N, Hunter DJ, Blenkinsopp EA (2009). Partners in health? A systematic review of the impact of organizational partnerships on public health outcomes in England between 1997 and 2008. J Public Health.

[25] Thomas J, Harden A (2008). Methods for the thematic synthesis of qualitative research in systematic reviews. BMC Med Res Methodol.

[26] Barnett-Page E, Thomas J (2009). Methods for the synthesis of qualitative research: a critical review. BMC Med Res Methodol.

[27] Popay J, Roberts H, Sowden A, Petticrew M, Britten N, Arai L (2005). Developing guidance on the conduct of narrative synthesis in systematic reviews. J Epidemiol Community Health.

