# Supplementary material for: Systematic review of the health and social determinants and outcomes of home cooking: protocol
Source: Syst Rev. 2015 Mar 28;4:35. doi: 10.1186/s13643-015-0033-3 (PMC4377200; doi:10.1186/s13643-015-0033-3)
Supplement: Additional file 1: — Sample search string for Ovid MEDLINE. Search string iteratively developed by information scientist. [file 13643_2015_33_MOESM1_ESM.docx]

**Additional file 1: Sample search string for Ovid MEDLINE - In-Process & Other Non-Indexed Citations and Ovid MEDLINE(R) 1946 to Present**

**Searched December 2014**

1. (dinner* or lunch* or breakfast* or meal* or food* or supper*).ti,ab.

2. ((home? or domestic*) adj2 (made or based or cook* or prepare or prepared or preparation)).ti,ab.

3. 1 and 2

4. (famil* adj3 (dinner* or lunch* or breakfast* or meal* or cook*)).ti,ab.

5. ((at-home or scratch or from-scratch or "put together") adj3 (dinner* or lunch* or breakfast* or meal* or food* or supper* or cook*)).ti,ab.

6. *cooking/ or exp meals/ or *food habits/

7. ((prepare or preparing or prepared or preparation or "make ready" or "make fit" or "put together") adj2 (dinner* or lunch* or breakfast* or meal* or food* or supper*)).ti,ab.

8. ((intake or consume$ or consumption) adj2 (fruit? or vegetable?)).ti,ab.

9. ((cook or cookery or cooked or cooking) adj5 (dinner* or lunch* or breakfast* or meal* or food* or supper* or fruit? or vegetable?)).ti,ab.

10. ((menu or food) adj2 (plan* or management)).ti,ab.

11. or/3-10

12. (attitude? or barrier* or behavio?r* or belief? or believ* or confiden* or deterrent? or effort? or habit? or influenc* or incentiv* or knowledge or practical* or practi?e* or self-efficacy or self-esteem or socio?economic* or responsibilit* or ritual* or routine? or motivat*).tw.

13. ((price? or cost? or expens*) adj3 (food* or fruit* or vegetable* or grocer* or produce)).tw.

14. ((financial or food) adj2 (secur* or insecur*)).tw.

15. ((women? or woman? or wive? or wife? or female or gender) adj2 (task? or role?)).tw.

16. ((culture or cultural) adj2 tradition?).tw.

17. (time adj3 (scarc* or constraint* or pressure? or availab* or organi?ing or organi?ation or plan* or prepar* or clean* or lack*)).tw.

18. (experience adj3 (cook* or prepar*)).tw.

19. ((lack* or limit*) adj3 (experience? or skill? or confidence)).tw.

20. socioeconomic factors/ or *self concept/

21. or/12-19

22. 11 and 21

23. ((better or improv* or enhanc*) adj3 (diet* or nutrition* or outcome* or sociab* or sociali?ation or self-esteem or "social determinant?" or survival or mortality or communication*)).tw.

24. ((reduc* or decreas* or improv*) adj3 (inequal* or obesity or bmi or "food insecur*" or portion? or calories)).tw.

25. ((better or improv* or increas* or enhanc*) adj3 (health or survival or mortality or "food secur*" or financ* or communication*)).tw.

26. (better or improv* or increas* or enhanc*).tw.

27. ((health* or social) adj3 (determinant? or outcome?)).tw.

28. (cook* adj2 (skill* or abilit*)).tw.

29. ((better or improv* or increas* or enhanc*) adj3 (((health* or social) adj3 (determinant? or outcome?)) or (cook* adj2 (skill* or abilit*)))).tw.

30. (esteem or confidence or self-efficacy).tw.

31. (cook* adj2 (skill* or abilit*) adj5 (esteem or confidence or self-efficacy)).tw.

32. ((better or improv* or increas* or enhanc*) adj5 outcome*).tw.

33. (health* adj2 (choose or choice?)).tw.

34. ((chang* or health*) adj2 (behavio?r* or habit*)).tw.

35. ((better or improv* or increas* or enhanc* or skill*) adj3 ((meal* or food) adj2 (manag* or plan*))).tw.

36. ((increas* or higher) adj3 (fruit* or vegetable* or vitamin* or nutrient*)).tw.

37. Long?term health.tw.

38. "disease prevention".tw.

39. (decreas* adj3 disease*).tw.

40. (health* adj3 (eat* or habit*)).tw.

41. (health* adj2 consumption*).tw.

42. ((reduc* or control) adj2 weight).tw.

43. ("lose weight" or weight?loss or "weight loss").tw.

44. (enjoy* adj2 (food* or cook* or fruit* or vegetable*)).tw.

45. or/23-25,29,31-44

46. 11 and 21 and 45
